# Challenges facing physicians in death certification of under-five mortality in Egypt

**DOI:** 10.1186/s12913-024-11780-9

**Published:** 2024-11-25

**Authors:** Mirette M Aziz, Nevein Dous

**Affiliations:** 1 Department of Public Health & Community Medicine, Assiut University, Assiut, Egypt; 2 Health Specialist in UNICEF Egypt CO, Cairo, Egypt; 3 AUC School of Continuing Education, Cairo, Egypt

**Keywords:** Death certification, Death notification forms, ICD_10, Health offices, Challenges, Egypt

## Abstract

**Background:**

Improving death certification of Under 5 Mortality (U5M) is an important pre-requisite for improving child health. This study aimed to obtain a clear and comprehensive understanding of the process of death registration of U5M and address the challenges of accurate certification faced by physicians at hospitals and health offices.

**Methods:**

A qualitative descriptive study was performed by conducting 10 Focus Group Discussions (FGDs) with physicians who are actively involved in the certification of U5M. Physicians were invited to participate from primary health care units, health offices, district and general hospitals in Cairo, Giza and Assiut Governorates. Field visits of health offices were performed to observe the procedure of death registration, visualize a sample of the hospitals’ Death Notification Forms (DNFs) and death certificates of U5M, and explore the used electronic system of death registration. Data analysis was performed using inductive thematic analysis.

**Results:**

The study clarified the procedure of death certification of U5M, starting from hospitals and ending at health offices. It was evident that there is a considerable deficiency in the system of registration and coding causes of death. Physicians of hospitals had a negative attitude regarding death registration. They were found to have deficient knowledge about standards of death certification. They had difficulty defining and reporting the chain of events leading to death. Poor diagnostic facilities hindered physicians from accurately reporting COD. Fear of legal accountability was a cause of avoiding detailed COD. For physicians in the health offices, other challenges were mentioned such as assigning junior physicians to manage the task of writing DNFs, poor quality of hospital reports and the unrealistic ICD-10 codes.

**Conclusion:**

Improving the accuracy of writing DNFs in hospitals is essential for providing reliable U5M statistics. Practical training of physicians, especially the junior ones, on ICD-10 classification and on identification and writing direct and indirect COD in the allocated lines in the death certificates is a priority. Establishing an audit system to assess the quality of the process of certification and updating the software of the electronic system for data entry would have a great impact.

**Supplementary Information:**

The online version contains supplementary material available at 10.1186/s12913-024-11780-9.

## Background

Mortality information and identification of the causes of death (COD) are important means of monitoring and assessing the level of health status of the population, prioritizing health issues, allocating budgets for health services, and directing research targets [1, 2]. Countries routinely invest significant resources into collecting mortality data from a variety of sources, including civil registration systems, health care facilities, health surveillance, and from other data sources such as censuses or household surveys. The primary purpose is to generate critical information to guide public health decision-making and plan the required health services [3]. Since 1990, the global Under-5 Mortality (U5M) rate has dropped by 59%, from 93 deaths per 1000 live births in 1990 to 38 in 2021 [4]. U5M, reliable data on COD is very critical, as it is the foundation for evidence-based informed health policy for planning and evaluating different public health interventions that are based on accurate child mortality estimates [5].

In-depth understanding of U5M causes in Egypt is essential for improving child health and achieving Egypt’s vision of Sustainable Development Goals (SDGs) 2030, which calls for reducing U5M rate to less than 15 per 1000 live births by 2030 [6]. However, monitoring of country progress towards achieving the SDGs is impossible without reliable mortality data provided by Civil Registration and Vital Statistics (CRVS) systems. Egypt has 27 governorates with 5471 local health units registering births and deaths. The National Information Center for Health and Population (NICHP) is responsible, in collaboration with the Preventive Sector, for the compilation and annual publication of the national statistics on COD, based on information reported in the death notification forms (DNFs) [7]. Egypt began using an electronic death filing system in 2013, aiming to the improvement of the accuracy of mortality statistics (Anecdotal evidence, Ministry of Health and Population (MOHP), 2023).

Accurate and correct application of the WHO guidelines for filling the death certificate form as well as correct coding of data according to ICD-10 standards are essential for the production of reliable mortality statistics [2]. However, it was observed that 21 out of 32 studies that examined the integrity of death certificates published in the last ten years reported an error percentage exceeding 90% [8]. In Egypt, a previous study recorded errors in 100% of DNFs, with 63.4% of DNFs contained major errors; underlying cause of death incorrectly attributed or placed in improper sequences [9]. Another study which assessed the trend of ill-defined COD in Egypt found that only around half of the deaths that occurred and were registered through the studied time series had the underlying cause of death declared [10].

Physicians’ knowledge and skills in correctly identifying the sequence of events leading to death is potentially the most significant contributing factor to the accuracy of death certifications [11]. However, the training on certification of COD is inadequate both in high income and low- to middle-income countries, and is often taught from the viewpoint of legal or forensic medicine, rather than emphasizing the public health importance of correct COD certification, resulting in suboptimal and biased COD information [2, 11].

For the year 2020, number of recorded U5M in Egypt was 44,300 deaths. The most frequently recorded causes of death were respiratory tract infection (32.4%), congenital anomalies (10.0%), prematurity (6.1%), bacterial infection (3.3%), accidents and poisoning (3.2%), diarrhea (3.0%), and malignancies (1%). Strikingly, 41% of mortality causes were recorded as “other unspecified causes” indicating poor quality of collected data (Anecdotal evidence from NICHP of MOHP).

We aimed in this study to obtain a clear and comprehensive understanding of the process of death registration of U5M. We also aimed to explore physicians’ perspectives towards reporting under five deaths in different Governorates of Egypt and identify the challenges of accurate death registration. We believe that this would be the first step for addressing the existing deficiencies for improving the death registration system.

## Methods

### Study design

A cross-sectional study using qualitative descriptive study design was performed. Focus Group Discussions (FGDs) approach was selected to obtain insight about physicians’ perspectives and experiences and because the group interaction usually enhance disclosure of the real life practices Also, in FGDs, participants tend to be influenced by group dynamics and individual responses [12].

### Data collection

 Recruitment of participants was done through the cooperation of the Primary Healthcare, Preventive and Curative sectors of MOHP, as physicians were invited through their health directorates. Physicians in active practice were invited to participate from primary health care units, health offices, district and general hospitals in three large Governorates; Cairo, Giza and Assiut Governorates. They were selected based on the recorded highest distribution of U5M in 2020 reports, and to represent different perspectives in different types of residence, with assumed different systems of registration. A purposive sample was used. For physicians working at hospitals, inclusion criteria were to be a pediatrician, in active practice, and directly involved in managing and reporting U5M. We also aimed to recruit physicians of a wide age and experience range, and of both sex.

Ten FGDs were performed; five FGDs were conducted with physicians working in health offices and the other five with physicians working in hospitals. Physicians of health offices were recruited from 15 health offices in Cairo, 9 health offices in Giza and 17 health offices in Assiut city at different districts and villages. We have combined elements of both data saturation and inductive thematic saturation, as data collection was continued till no additional data and no more codes or themes were found. All discussions were recorded and transcribed. Each FGD lasted between 90 and 120 min.

### The study FGD guide

Data was collected through semi-structured discussions guided by a list of open questions. In addition, probes and follow-up questions were used for better and deeper understanding of the participants’ answers and to encourage them to talk freely about their experiences and to add their suggestions and recommendations.

Prior to conducting FGDs, field visits were performed to two health offices in order to observe the flow of work and the procedure of death registration. Informal discussions were also performed with the clerks and physicians in the health offices to help the researcher develop the FGD guide, in addition to visualizing a sample of the hospitals DNFs and death certificates of U5M and exploring the used electronic system of registration and process of coding and data entry.

Two different FGDs guides were developed; one for physicians working at hospitals and the other for physicians working at health offices (Supplementary file 1 and 2). FGD guides were designed to explore the frequent U5M causes in practice, physician’ ability to diagnose COD, interaction between the family, hospitals and health offices, the usual procedure for issuing death certificate, physicians’ attitude regarding accurate death certification, challenges to maintaining accuracy of registration. Causes of U5M mentioned in NICHP, 2020, and the unexpected high frequency of “other causes” were also discussed. An empty DNF was distributed for discussing its items and how it is filled up by physicians. ICD-10 codes were also distributed during the meetings for exploring physicians’ familiarity with its classification and their opinion regarding its use. Received training and training needs were also discussed.

### Qualitative data analysis

Data analysis was performed using inductive thematic analysis. The transcripts were read repeatedly and the raw data was coded thematically. Annotations were made and key phrases and words were highlighted, cut out, and sorted according to themes. Codes and labels were attached to text parts related to a specific theme, leading to a set of descriptive themes and subthemes per transcript. Then, all codes were clustered into themes and subthemes. Themes were confirmed, modified, or discarded from ongoing analysis by re-examination of earlier data and during subsequent data collection (Table 1).

## Results

### Demographic characteristics of the study participants

The FGDs were conducted with 120 total participants (67 physicians of health offices and 53 pediatricians); 63% of participants were males and 37% were females. They had different levels of experience, as the groups of hospitals physicians included residents, assistant specialists, specialists and consultants. In health offices, the experience also ranged from the newly graduated physicians in primary health care units to physicians with more than 20 years of working experience in health offices. This diversity allowed for obtaining in-depth information from different backgrounds and capturing the insight and recommendations at all levels.

### Process of death registration

The discussions performed in this study in addition to the field visits to the health offices have clarified the system of death registration in Egypt. The process of death registration is complementary between hospitals and health offices. Physicians working in hospitals report U5M cases that occur in hospitals by writing a hospital medical report kept in the hospital and a DNF transmitted to health office for coding and electronic documentation. For deaths that occur at home, out of health facilities or brought dead to the hospitals, cases are reported by the relatives to the health office directly, where physicians of health offices inspect the corpse and exclude a medico-legal cause of death or refer to forensic medicine. In all cases, physicians of health offices are responsible for coding COD, recording it on the electronic system and issuance of the death certificate.

### Challenges of accurate death certification at hospitals

Most physicians described a high level of inaccuracy in the process of death certification of U5M. Accuracy of registration was found to be associated with many challenges, yielding inaccurate statistics for effective health planning.

#### Negative attitudes of physicians towards death registration

Most physicians believed that filling out a DNF is performed as routine paper work, only for obtaining the burial permission. The majority of participating physicians believed that it is neither actually required to be accurate, nor it would help in any improvement of the health system. Most of them thought about this issue out of a clinical perspective, not a public health perspective.

**Female pediatric resident, general hospital, Cairo**:

*“I am not really concerned about this issue*,* I have nothing to do with accuracy of registration*,* with my respect*,* but I manage my case*,* that’s what I care about*,*”*

Physicians working in different hospitals have also mentioned that they might be very meticulous in writing a detailed patient record and death forms that are kept in their hospital to safeguard against any formal or legal account. These forms include all details since admission including patient history, results of examination, investigations performed, diagnosis, medication taken, operative interventions and a report illustrating causes of deaths. However, out of their belief that DNF is actually not important, dead cases are usually discharged with only a DNF that is reporting only the direct COD, which is inaccurately recorded in the majority of cases.

**Female pediatric consultant, general hospital, Giza**:

"The hospital report is very detailed,, it includes the cause of death, child’s condition on admission, all the investigations and procedures performed,, all complications, but this form is only for the hospital."

**Male physician, district hospital, Assiut**:

"Cases may die at the hospital and discharged without any reports,, yes,, I’ve worked for two years now in the hospital and I’ve witnessed that,, about 70 to 75% of cases are discharged [on demand], without any document."

#### Poor physicians’ knowledge regarding death certification standards

Almost all physicians mentioned that they did not know that the underlying causes of death (UCOD) should be written. They believed it was very sufficient to write the direct COD in the DNF, as it would be the only required for issuing a death certificate.

**Male pediatric consultant, district hospital, Assiut**:

*“The physician at health office would finally search for the direct cause of death*,,* he would not need the underlying causes that have led to death*,,* for example*,* a case of pneumonia would finally ends up in cardiac arrest*,* so*,* I only write “cardiac arrest”*.

Writing the direct COD only results in underestimation of many causes of U5M. The direct COD is mostly a complication of the underlying causes or describes the mechanism of death. For example, physicians mentioned that gastroenteritis are usually recorded as “septic shock”, as a direct cause of death, without mentioning that gastroenteritis was the underlying cause, meningitis as “disturbances in conscious level”, prematurity as “respiratory distress”, pneumonia as “heart failure”,,,etc.

**Female pediatric specialist, general hospital, Cairo**:

"We observe many cases of gastroenteritis, but only dehydrated and shocked cases die,,, so we don’t record as “gastroenteritis”, but as “septic shock”, because this is the final diagnosis, some babies may also die because of aspiration caused by “gastroenteritis associated vomiting”, I’ve seen that actually,, such cases are recorded as “aspiration” as the cause of death,, he didn’t die actually because of gastroenteritis or dehydration"

Most physicians, especially those who did not have a previous experience of working in health offices, also had very deficient information about the subsequent registration procedures of the DNF at the health offices. They did not know that this form would be used for registration of death causes in the NICHP of MOHP, using ICD-10 codes. The lack of knowledge about the electronic registration system has been referred to as an important cause of the mentioned perspective of inaccurate registration.

**Female physician, health office, Assiut**:

"Those physicians who have not previously worked at a health office don’t know about the existence of an electronic registration system,, so they write anything,, they believe it is the end,, I don’t then find a diagnosis to insert,, a clear and definite cause,, even in reports from the university hospitals,, they don’t know the next steps after writing his report,, they think it is just for obtaining the burial permission and death certificate"

Most physicians reported that they write the DNF in the form of a paragraph describing the medical condition of the dead case, the important interventions performed and whether the case was subjected to resuscitation and then the cause of death as cardiac or respiratory arrest. They don’t usually use the assigned lines in the DNF form for the direct, indirect and contributory causes of death. They may also write multiple non sequential causes per line.

**Male physician, general hospital, Giza**:

"In most cases we write that the child had so and so,,, then resuscitation was performed and he finally died,,, we write the final diagnosis, we write it in the form of a paragraph, not in the way you are talking about [refers to writing the chain of events leading to death]"

This practice creates a major difficulty for most physicians working at health offices to extract a direct and an indirect COD from the DNFs delivered from hospitals in order to insert in the electronic system of death registration.

They have attributed their poor knowledge regarding death certification issues to the fact that they did not have a relevant training. They usually receive trainings related to their clinical specialty, with negligence of these topics, which they view as tasks or the responsibilities that are not of their role and more related to physicians in administrative positions.

#### Poor diagnostic facilities in hospitals

When ICD-10 codes were distributed in the meetings, most physicians expressed their annoyance on reading such classification, as most codes included causative organisms, stages of the disease and difficult differentiation. They commented that these codes were so many to explore and use. They also mentioned that they usually face limited common causes that lead to U5M in their practice, which do not require such complicated classification. They also referred to the poor diagnostic facilities, especially in district hospitals of MOHP with limited resources, which add to the difficulty of using such codes, especially when there is no time for referral to a higher level of health facilities to complete the required investigations. Some physicians mentioned that even some basic laboratory and radiological facilities are deficient in their place of work, which creates difficulty in reaching an accurate diagnosis of many causes mentioned in the ICD-10.

**Female physician, general hospital, Assiut**:

“*ICD causes need performing sophisticated investigations*,,* I don’t have these diagnostic facilities in the hospital to reach such required accurate diagnosis*,* for example; pneumonia could be viral or bacterial*,* how would I differentiate in absence of culture and CBC? Which classification of all those mentioned in the ICD codes should I select?”*

#### Poor history of cases presented at emergency reception or brought dead to hospital

Most physicians mentioned the high degree of uncertainty about causes of death. This was observed especially for cases brought in critical conditions from home or cases of death on arrival. Poor documentation from the families and inaccurate history, especially in case of poor economic and educational levels adds to the uncertainty about causes of death. Families may be also dishonest regarding any harmful exposure.

**Male pediatric specialist, general hospital, Cairo**:

"There are cases where you never know what have happened before what,, there are a dark zone that you never discover, some cases are really obscure,, I cannot say that I can always identify the cause of death. The family do not have information on and cannot help use with details in most cases,,I’ve recently observed a preterm case,, he died in the first 24 h,, was it because of pneumonia? Sepsis?? Other causes? I did not have enough time to investigate,,"

#### Physicians have concerns related to legal accountability

A considerable proportion of physicians have mentioned that they may not record the accurate cause of death in the DNFs that should be sent to the health offices, intentionally, as these forms are handed to the child family, who may accuse the physicians of being responsible for the death of their children, through the occurrence of inevitable complications on admission, such as nosocomial infection, anesthetic or surgical complications. Despite being considered, in the field of medical practice, as acceptable sequel of the admitted child health conditions, most physicians perceived that families may use such causes for charging the physicians, especially that most families have poor or no medical knowledge.

**Male pediatric resident, general hospital, Cairo**:

*“We always try to keep it non-specific*,* to be in the safe side*,,*if the official documents that are sent to health offices include the real causes*,* such as any “nosocomial infections”*,,* “a complication of surgical intervention”*,,,*this will cause problems that we are not in need of*,,* if the patient’s family has a document that their child died because of infection that he got in the hospital*,* they would surely make troubles*,,,* so we don’t write such causes*,,* especially that families would not understand that these are expected medical complications “*.

### Challenges of accurate death certification at health offices

#### Insufficient training of newly graduated physicians

A highly documented challenge mentioned by physicians of health hospitals is that newly graduated physicians are usually assigned to work at primary health care (PHC) units and health offices before being able to apply for residency. At PHC units, the General Practitioners (GPs) are responsible for the duties of health office of the unit in addition to the outpatient clinics work. Physicians mentioned they may have some clinical experience, gained during working as health officers before graduation, but they face the tasks of death certification for their first time in life when assigned to perform them. They complained of not having practical formal training on the standards of certification, and a few of them who had mentioned having a previous training, described the training to include administrative issues and the legal aspects of cases documentation, rather than emphasizing the public health importance of correct COD certification, introducing the required DNF, how to fill out, how to identify the UCOD and to select the proper ICD-10 classification and using the electronic system of registration, leading to their non-familiarity with the required system.

**Male pediatric resident, district hospital, Cairo**:

"When I was working in the health office,, we used to choose [not elsewhere classified] codes, as we don’t know the other codes, we have the down list but we don’t know how to search and reach the correct diagnostic classification,, it is really difficult, I knew about 20 causes that I usually used to select from,, I think it is a matter of fate and destiny, the cause is not important."

**Male physician, health office, Assiut**:

"The main cause of the non- familiarity with the system that we have not received a relevant training ,, we should use a system that we know nothing about,, how should I insert causes, while not trained on ICD-10 codes?"

#### Poor quality of hospital reports

Receiving poor quality DNFs from hospitals was a major daily experienced challenge. Physicians of health offices have attributed the unclear reports either due to insufficient experience of residents who usually work in the emergency reception or to not considering the importance of such forms by hospitals. They mentioned that “Cardiac and respiratory failure” are the most frequent found COD mentioned in hospital reports, leading physicians of health offices to ask families and insert the code of their own conclusion, which would be inaccurate and non-specific in most cases.

**Female physician, health office, Giza**:

"Physicians of curative sector don’t provide us with details about the case, they think that such data is unnecessary for physician of health office, we usually receive ambiguous reports from the hospitals, so the physician in the health office asks the family about the cause and write what they mention, which is mostly incorrect,,"

**Male physician, health office, Cairo**:

"Our main problem is that 90% of causes reported by hospitals is written by the residents or house officers who were in the shift,, what do you think he would write?? we always try to conclude the cause from the hospital report, so we may write cardiac failure, as it is the most logic cause that we know, but it is not the real cause,, we know,,, the real cause is always missing"

However, physicians of health offices have cleared their side from being responsible for the inaccurate registration, due to the deficient reports from hospitals. Physicians of health offices explained that it is not their role to conclude the direct and indirect COD out of a poorly written vague document sent from hospitals. They mentioned that their role should be restricted to copying the diagnoses mentioned in such cases, and just coding appropriately, as they are examined by a credible health setting. In addition, they have no formal communication with hospital physicians to ask for clarifying the ambiguous DNFs.

**Male physician, health office, Cairo**:

"I should not be asked to discover the cause of death,, I did not examine the case, so do not blame me,, I said this hundred times,, ask the physicians in hospitals to write good quality reports,, when I receive a cause of death of a child admitted in cancer institute as “cardiac and respiratory arrest”, what does this mean? Why was he admitted in the cancer institute? We finally write the causes we receive,,"

Some physicians have also attributed the poor quality of hospital reports to the lack of any form of supervision on these reports writing before being given to the family to deliver to the health office. Physicians of health offices themselves have never received any feedback on all the incorrectly inserted ambiguous and unspecific codes.

**Female physician, health office, Cairo**:

No,, we have never received any audits,, even when we submit non- specific codes, the physician can only discover the errors before certification,, nothing other than that,, there aren’t any monitoring or follow up,,

Physicians working at hospitals have also mentioned the existence of a death committee in the hospital, which role in inactivated, without any form of external or internal auditing, adding to the poor interest in performing accurate reporting.

**Male physician, general hospital, Assiut**:

"We have a death causes committee at the hospital, from 3 years ago, but it is virtual,, we are supposed to have a meeting and discuss causes of death with the head of department, this is important in order to avoid these causes thereafter,,"

#### Unrealistic ICD-10 codes

An important issue has been addressed by physicians of health offices, and which has been also mentioned in the discussions with pediatricians, is the classification used in ICD-10 coding. The existence of all these unusable codes, as they described, makes it difficult for coders to select the proper code and may lead to selecting “unspecified category” to avoid the created confusion.

**Female physician, health office, Assiut**:

“ *We should update the system by inserting only the codes which are common in use*,,* not all the codes*,,* for example pneumonia or gastroenteritis*,,* but not the very specific codes that we would never know*,,* I may write prematurity as a cause of death but none of the underlying sophisticated classification”*.

## Discussion

Despite the importance of accurate death certification, errors in the completion of the medical certificate of COD by physicians have been documented globally, with as many as half of all registered deaths do not have an accurate COD assigned [13]. In low and middle-income countries, causes of U5M are frequently inaccurately registered by the CRVS, and nearly half of these countries fail to meet the United Nations standards for death registration [14]. In this study, we aimed to address the challenges of accurate U5M certification faced by physicians at hospitals and health offices in Egypt.

It was found that death certification has many deficits and the accuracy of which faces major challenges. We found that the main problem emerges in hospitals, which are supposed to be an accurate source of mortality data, as it is expected that hospital physicians are able to correctly identify patients’ underlying COD, since hospitals usually have clinical protocols for diagnosis and monitoring disease progression [15]. However, ambiguous DNFs written by physicians of the hospitals had made it difficult for physicians of health offices to extract a clear COD to be coded and reported accurately. In line with our findings, hospital death certificates have been shown to be of poor quality in several countries, such as Philippines and Bangladesh [5, 16, 17] where physicians failed to adhere to international standards in completing the medical certificate. Moreover, a systematic review which studied the accuracy of COD in hospital data reported substantial misdiagnosis of COD [18].

Errors in hospitals completion of DNF were attributed to several reasons. The most important of which was the negative attitude of physicians regarding the importance of these forms. Participants of our study perceived DNF as routinely performed paper work that have no impact on improving health systems or making interventions. Their negative attitude was reflected in the careless filling out of DNFs transferred to health offices, while maintaining accuracy of reports kept in hospitals [8].

Moreover, physicians described their poor knowledge regarding death certificate guidelines and ICD coding system. Most of them did not know that UCOD is required to be recorded in the DNFs. They believed that the direct COD is all what matters. In line with our findings, a previous study in the Kingdom of Bahrain, found that over two-thirds of physicians were unaware of the death certificate completion guidelines and the majority (97.2%) did not know of the ICD coding system [19]. The physicians’ lack of attention to the record of underlying COD has been also reported in other studies [5, 17].

However, negative attitude and poor physicians’ knowledge are not always the drivers of inaccurate reporting. Some physicians mentioned that they believe in the importance of these forms for vital registration and national statistics, but they experienced difficulty in determining the UCOD and complained of uncertainty about COD. It was found that uncertainty is commonly observed in several vital registration systems of different countries [19–21], leading physicians to use certain terms such as. “pneumonia” or “myocardial infarction” [22]. It has been also documented recently in Egypt in a study conducted in Alexandria governorate [23], where the percentage of the undetermined causes of death was 23.0%. Moreover, insufficient data obtained from interviewed relatives, especially in case of home deaths adds to the existing uncertainty.

Ill-defined or vague conditions of death should not be reported as UCOD, as they provide little information to guide proper interventions [17]. However, ill-defined causes were frequently mentioned to be reported in the DNFs. Reporting ill-defined COD were also a major challenge identified by previous studies, where ill-defined or unknown COD represented more than a quarter of reported causes [24, 25]. Similar to previous studies in UK and USA [26, 27], we found a general tendency among physicians to assign the cause of death to “cardiac failure”. We found that overstating of cardiovascular causes of death in our study was either because physicians did not know that it is a “mechanism” not a “cause” of death or as an exit out of hesitancy in case of uncertainty about the real COD.

An important finding that worth addressing is the lack of supervision on writing DNFs in hospitals and on selecting the proper codes by health offices physicians. Absence of auditing give the physicians the sense of security regarding making errors, in their interpretation of the correct clinical diagnosis or in writing their diagnoses into the DNF [5]. This finding complies to the finding of a previous study in rural Bangladesh which found that hospital physicians failed to adhere to the death certificate completion guidelines until a departmental head emphasized these guidelines [17] .

Another important theme was created, which is avoiding details in order not to be questioned for responsibility. This perception could be attributed to the observed changes in the patient–doctor relationship nowadays, as doctors are no longer regarded as infallible and beyond questioning. Unfortunately, a malpractice case can ruin the doctor’s career and practice [28]. Physicians have complained that they feel insecure all the time regarding being accused of negligence and malpractice, especially as the DNFs are handled to the relatives who have no medical background and may perceive the written causes as physicians are accused of their child death.

Another overlooked challenge is the content of ICD-10 codes, which has been also addressed recently [29, 30], as many codes included in ICD-10 classification have little or no public health value because they are too vague, are an immediate or intermediate COD, or cannot explain UCOD and has been termed “Garbage coding”, as they are truly unhelpful for policy and are used frequently by physicians to certify deaths, distorting mortality statistics. Strategies to improve COD data quality in hospitals should address the unusable and insufficiently classified codes and reduce them, being more realistic about countries’ diagnostic capacity according to its level of development.

Most physicians largely attributed poor knowledge and non- familiarity with standards, whether of documenting or coding the UCOD according to ICD-10, to insufficient experience of resident and junior physicians, in particular, as the most junior physicians are commonly assigned the tasks of maintaining clinical records and of writing DNFs. Beginner practitioners and residents were found to make errors in completing death certificate forms in several studies [31, 32]. This was attributed to not receiving appropriate practical training on writing death certificates and ICD-10 codes in the beginning of practice. It is worth mentioning that training would have great impact, as previous studies documented a marked decrease in errors when brief educational interventions such as workshops, didactic seminars or distribution of printed guidelines [27, 33].

**Table 1:** Description of emerging themes

**Main theme**	**Subtheme**
Challenges of accurate death certification at hospitals	Negative attitudes of physicians towards death registration
	Poor physicians’ knowledge regarding death certification standards
	Poor diagnostic facilities in hospitals
	Poor history of cases presented at emergency reception or brought dead to hospital
	Physicians have concerns related to legal accountability
Challenges of accurate death certification at health offices	Insufficient training of newly graduated physicians
	Poor quality of hospital reports
	Unrealistic ICD-10 codes

### Strengths and limitation of the study

This is the first study that explored the death certification system of U5M in Egypt from the public health perspective, not from the forensic perspective, providing practical implications for improving policy based on needs assessment. Conducting the study in different governorates with different socio-demographic and cultural characteristics ensured the unity of the faced challenges at different levels. Moreover, being academic researchers, not related to the administrative sector of the participants, and the qualitative nature of the study with keeping anonymity provided participants with a sense of confidentiality, facilitated disclosure, and increased the credibility of the findings. However, being a qualitative study, the findings cannot be assumed to apply to all physicians involved in death certification, but depends solely on the experience of the participants and the skills of the moderators.

### Implications

It was concluded from the study that integration between the Primary healthcare, Preventive and Curative sectors is the key for improving the certification of U5M. The Curative Sector should work on improving the accuracy of writing DNFs in hospitals, as it is the key for providing reliable mortality statistics. Accurate DNFs will enable the physicians at health offices to properly code the COD without resorting to unspecific and undefined causes. This could be achieved through practical training of physicians, especially the junior ones, on ICD-10 classification and on identification and writing direct and indirect COD in the allocated lines, one cause per line. Continuous training should be provided for health officers, newly graduated and in the first year of residency. Establishing an audit system with specific key indicators to assess the quality of the process of certification, with providing active feedback for revision and correction, would have a great impact. It is also recommended to establish direct electronic communication between hospitals and health offices for sending all the required detailed data of the dead case. Updating the software of the electronic system for data entry should be performed with reducing the “Garbage codes”, and including only the causes that are directly related to causing death, for more accurate selection and submission of accurate codes.

## Supplementary Information

Supplementary Material 1.

Supplementary Material 2.

## Data Availability

The data used and/or analysed during the current study is available from the corresponding author on reasonable request.
