# Supplementary material for: Challenges facing physicians in death certification of under-five mortality in Egypt
Source: BMC Health Serv Res. 2024 Nov 25;24:1459. doi: 10.1186/s12913-024-11780-9 (PMC11587660; doi:10.1186/s12913-024-11780-9)
Supplement: Supplementary file 1 — Supplementary Material 1. [file 12913_2024_11780_MOESM1_ESM.docx]

**
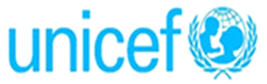
**

**Focus group discussion guide**

**Participants:** Physicians working at Health offices

Greetings of the day,, today we need to discuss with you an important issue, which is under 5 mortality (U5M). We need to benefit from your experience in recording U5M at health offices and identify the challenges that you might be exposed to in accurate registration. You have been invited to participate because of your experience of working at MOHP health offices.

Please, everyone, introduce yourself; name, age, qualifications, years of experience, and place of work.

**Rules for discussion:**

- There is no correct or wrong answer, but the discussion needs to be organized with giving everyone the opportunity to express his thoughts, opinions and perspectives.
- The discussion is audiotaped, but the tapes will be with the research only, with keeping the privacy and anonymity of all participants.
- The transcripts will not include names or any personal data of the participants

Any question?

1. We would like to understand the system of death registration at health offices, what are the tasks of physicians? What are the tasks of clerks?
2. Do you think it is the role of the physician to record the cause of death and insert data in the electronic system of death registration?
3. What are the challenges that physicians might face for accurate death registration at heath offices? What are the specific challenges related to U5M?
4. What are the most frequent causes of U5M you usually record at health offices? in the first month? In the first year?
5. Where does U5M usually take place? At home or do you usually receive a DNF from the hospital where the child died?
6. Do you find the DNF received from the hospital accurate and properly written? Is it easy for you to extract the direct and underlying causes of death out of it?
7. When do physicians of health offices have to write DNF themselves?
8. What are the procedures performed when the health office is notified by U5M at home?
9. Is it easy to determine the cause of death in case of U5M? Why?
10. Do families support physicians of health offices by information related to the medical history of the child?
11. How do you fill in the DNF? Do you use the assigned lines for direct and underlying causes of death? Or do you only write that the death was normal or not normal?
12. Is accurate registration important or only routine paper work?
13. We have observed that cardiovascular causes represent a great proportion of cause of death of under 5 children? How do you explain that?
14. Do you consider “Cardiovascular and respiratory arrest” as causes or mechanisms of death?
15. Do you face cases of U5M in which the cause of death is difficult to be determined? How do you record the cause of death then?
16. What do you know about ICD-10 codes? Do you use ICD-10 coded in recording causes of U5M? Do you know how to use these codes?
17. Have you ever been trained on death certification and writing the DNFs? What was the content of the training? Did it add to your experience? What was deficient in this training?
18. What are your suggestions to improve the system of death registration of U5M at health offices?

**Thank you for your time and cooperation,,**
