# Supplementary material for: Challenges facing physicians in death certification of under-five mortality in Egypt
Source: BMC Health Serv Res. 2024 Nov 25;24:1459. doi: 10.1186/s12913-024-11780-9 (PMC11587660; doi:10.1186/s12913-024-11780-9)
Supplement: Supplementary file 2 — Supplementary Material 2. [file 12913_2024_11780_MOESM2_ESM.docx]

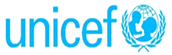


**Focus group discussion guide**

**Participants:** Physicians working at MOHP hospitals

Greetings of the day,, today we need to discuss with you an important issue, which is under 5 mortality (U5M). We need to benefit from your experience in dealing with U5M with different causes and circumstances. You have been invited to participate because of your experience of working at MOHP hospitals.

Please, everyone, introduce yourself; name, age, qualifications, years of experience, and place of work.

**Rules for discussion:**

- There is no correct or wrong answer, but the discussion needs to be organized with giving everyone the opportunity to express his thoughts, opinions and perspectives.
- The discussion is audiotaped, but the tapes will be with the research only, with keeping the privacy and anonymity of all participants.
- The transcripts will not include names or any personal data of the participants

Any question?

1. We would like to know the most frequent causes of U5M faced at your place of work? What are the frequent causes in the first month? In the first year?
2. Where does U5M usually take place? At home? Pediatric departments? Intensive care units? Operating theater?

**The statistical report of U5M causes of death, 2020 is displayed for discussion**

1. We have observed that cardiovascular causes represent a great proportion of cause of death of under 5 children? How do you explain that?
2. Do you record cardiovascular or respiratory arrest as causes of death in the death notification forms (DNFs)? Do you consider these as causes or mechanisms of death?
3. Do you face cases of U5M in which the cause of death is difficult to be determined? How do you record the cause of death then?
4. What are the procedures performed after the child death at the hospital?
5. What about “death on arrival”?
6. Who writes the death notification form? Should it be the physician attending the case? Could anyone else perform this duty? Could the nurse?
7. Would you discharge dead cases from the hospital without DNFs?

**The DNF is displayed for discussion**

1. Is this the used DNF by all hospitals?
2. How do you fill in the DNF? Do you use the assigned lines?
3. Do you know how to classify the causes of death as underlying cause of death, direct cause of death? Contributory cause of death?
4. Do you consider the accurate registration of this form important?
5. Would you ever record, intentionally, an inaccurate cause of death? What could be the drivers to do that?
6. What do you know about ICD-10 codes? Do you use ICD-10 coded in recording causes of U5M? Do you know how to use these codes?
7. Have you ever been trained on death certification and writing the DNFs? What was the content of the training? Did it add to your experience? What was deficient in this training?
8. What are your suggestions to improve the system of death registration of U5M at hospitals?

**Thank you for your time and cooperation,,**
